# The Effect of Low Monotonic Doses of Zearalenone on Selected Reproductive Tissues in Pre-Pubertal Female Dogs—A Review

**DOI:** 10.3390/molecules201119726

**Published:** 2015-11-19

**Authors:** Magdalena Gajęcka, Łukasz Zielonka, Maciej Gajęcki

**Affiliations:** 1 Department of Epizootiology, Faculty of Veterinary Medicine, University of Warmia and Mazury in Olsztyn, Oczapowskiego 13/01, Olsztyn 10-718, Poland; 2 Department of Veterinary Prevention and Feed Hygiene, Faculty of Veterinary Medicine, University of Warmia and Mazury in Olsztyn, Oczapowskiego 13/29, Olsztyn 10-718, Poland; lukaszz@uwm.edu.pl (Ł.Z.); gajecki@uwm.edu.pl (M.G.)

**Keywords:** zearalenone, reproductive system, pre-pubertal female dogs

## Abstract

The growing interest in toxic substances combined with advancements in biological sciences has shed a new light on the problem of mycotoxins contaminating feeds and foods. An interdisciplinary approach was developed by identifying dose-response relationships in key research concepts, including the low dose theory of estrogen-like compounds, hormesis, NOAEL dose, compensatory response and/or food tolerance, and effects of exposure to undesirable substances. The above considerations increased the researchers’ interest in risk evaluation, namely: (i) clinical symptoms associated with long-term, daily exposure to low doses of a toxic compound; and (ii) dysfunctions at cellular or tissue level that do not produce clinical symptoms. Research advancements facilitate the extrapolation of results and promote the use of novel tools for evaluating the risk of exposure, for example exposure to zearalenone in pre-pubertal female dogs. The arguments presented in this paper suggest that low doses of zearalenone in commercial feeds stimulate metabolic processes and increase weight gains. Those processes are accompanied by lower proliferation rates in the ovaries, neoangiogenesis and vasodilation in the ovaries and the uterus, changes in the steroid hormone profile, and changes in the activity of hydroxysteroid dehydrogenases. All of the above changes result from exogenous hyperestrogenizm.

## 1. Introduction

This review paper was motivated by the need to address problems that surfaced in our previous research into pre-pubertal female dogs exposed to zearalenone (ZEN). We set out to investigate whether animals should be exposed to high doses of undesirable/harmful substances in toxicological studies [1]. Attempts were also made to determine whether mycotoxins (undesirable substances, but not xenobiotics) (i) should be administered at NOAEL (no observable adverse effect level) doses in view of the fact that mammals and humans learn to tolerate low monotonic doses of toxins which are administered for long periods of time [2]; (ii) can be used by the body to meets its basic needs [3,4,5]; (iii) can produce therapeutic effects [6]. Our aim was also to explain whether the observed changes in the health status of animals are indicative of mycotoxicosis or result merely from the exposure to undesirable substances.

Female dogs are a good model for our research because those monogastric animals are not phylogenetically adapted to ingest plant-based feeds contaminated with mycotoxins. Most commercial feeds are based on cereals, and many of them contain mycotoxins, including ZEN [7]. Female dogs can be expected to respond rapidly and unambiguously to the presence of ZEN in feed, even at NOAEL doses. At present, there are no methods that guarantee complete elimination of undesirable substances, including mycotoxins (detoxification, destruction of mycotoxins), from plant-based feeds. Another question that arises in connection with the above is whether complete elimination of mycotoxins from plant materials is an absolute necessity. In the available literature, the above questions were rarely addressed by studies of female dogs.

The observations made by Waters [8] provided a wider perspective on reproductive problems in female dogs. Age-related changes in the reproductive system of female dogs should be analyzed from the moment of sexual maturation when reproductive organs become exposed to various risk factors including undesirable substances, such as ZEN which is ingested with commercial feed. Zearalenone is an important epidemiological factor, and even small doses of ZEN can induce considerable changes in endocrine and intracrine processes [6] and influence homeostasis in the reproductive system from birth till death.

The presented hypotheses are validated by the results of few previously published studies [9] where female dogs were exposed to high doses of ZEN over short periods of time. Changes in the animals’ reproductive system, mainly in the ovaries and the uterus, were observed in those studies and in evaluations of metabolic profiles of multiparous mixed-breed bitches [10]. Histopathological changes in the ovaries included degeneration of cells and tissues and inhibition of their biological activity. Edema and extravasations were observed in the uterus of a female dog exposed to a ZEN dose of 200 μg/kg BW for 7 days. In other studies, the loss of junctions between granular cells and the formation of intercellular spaces pointed to the loss of regulatory functions of the granular layer. Cell populations were visibly differentiated. In female dogs, exposure to ZEN intensified metabolic processes in the circulatory system and the liver. It should be noted, however, that the referenced studies included highly generalized analyses of entirely different experimental materials.

The discussed findings were not validated by studies where animal and human subjects were exposed to low doses of undesirable substances over long periods of time. The results differed considerably from those reported during exposure to with high doses of such substances. The observed variations involved an absence of clinical symptoms of toxicity, but changes (stimulation or compensatory response [11]) were noted at cellular or tissue level. The above variations necessitate the search for new information to assess safety and risk in the decision-making process [12].

Our knowledge of mycotoxins, substances that cause various disorders (mycotoxicosis) in humans and animals depending on the administered dose, period of exposure and metabolic rate, remains limited [13,14]. Mycotoxins are secondary metabolites produced mainly by fungi of the genera *Fusarium*, *Aspergillus* and *Penicillium* during their growth and development. Fungi can produce one or more secondary metabolites, not all of which have toxic properties. Mycotoxins reach the body in various concentrations, and most of them are ingested with feed or food [15]. They exert hepatotoxic and nephrotoxic effects, and they also have carcinogenic, mutagenic and teratogenic properties. Estrogenic mycotoxins cause reproductive disorders in livestock [16], companion animals [10] and wild animals [17].

Mycotoxin-producing fungi of the genus *Fusarium* are most widely distributed in all climate zones. The mycoestrogen ZEN is one of the major mycotoxins with estrogenic properties [18]. ZEN is non-steroidal estrogenic mycotoxin and a specific hormone regulating the sexual reproduction of *Fusarium* species (sexual stage–*Gibberella zeae*) [19].

Mycotoxicosis have been long studied in humans and animals [15], and they pose a serious health concern. Zearalenone mycotoxicosis in livestock [20] and companion animals [21] lead to developmental and reproductive problems and cause economic losses. Dogs are among the most popular species of companion animals. Female dogs are seasonally monoestrous animals which are relatively often diagnosed with reproductive disorders [21]. Species-specific hormonal regulation of reproductive processes in bitches, which involve long proestrus and estrus stages, long progesterone and prolactin cycles, and high sensitivity to endogenous and exogenous estrogens [22], could play an important role in the etiopathogenesis of those disorders. The administration of hormones to female dogs for therapeutic or biotechnological (contraceptive) purposes is another important consideration [23].

The potential effects of feed-borne mycotoxins (mycoestrogens) on animals have been completely disregarded to date [24]. Preliminary studies revealed high and varied levels of ZEN in commercial feeds [25]. Zearalenone was identified in 42 out of 45 analyzed dog feed samples in concentrations of 5.0 to 299.5 μg/kg of the product [26]. Dogs are often fed monodiets for long periods of time, and ZEN contamination could have particularly harmful effects on this species of companion animals. Zearalenone is an estrogenic mycotoxin and an endocrine disrupting compound [3,5], and female dogs are particularly sensitive to estrogens. Elevated concentrations of endogenous and/or exogenous hormones (leading to hyperestrogenizm relative to the animal's physiological condition) also increase the risk of other systemic disorders. Long-term exposure to feeds contaminated with ZEN can disrupt hormonal regulation of reproductive processes [5] and lead to ovarian [27] and uterine dysfunctions [21]. The ovaries and the uterus are particularly sensitive to estrogenic substances which can contribute to permanent changes associated with ovarian cysts and/or spontaneous endometriosis [23]. ZEN can thus influence two types of structures: (i) organs targeted by tropic hormones; and (ii) functionally changed tissues and cells, which can lead to changes in other organs or in the entire body.

For the above changes to take effect, ZEN has to come into contact with the gastrointestinal tract which is the first barrier protecting the body against undesirable substances that are present feed [14]. Zearalenone penetrates the intestinal wall, enters the bloodstream and is distributed throughout the body where it can provoke various changes, not always pathological, or remain neutral [28], subject to the dose. In the intestines, this mycotoxin undergoes biological transformations, and those processes can begin already in the plant to produce compounds more toxic than the parent substance [4]. The final effect is always determined by the dose [29].

Doses below NOAEL values (the highest dose that does not induce clinical symptoms) produce completely different local and systemic symptoms than those noted during mycotoxicosis provoked by doses higher than NOAEL [30,31,32].

In recent years, the traditional dose-response relationship has been undermined by the low dose hypothesis, in particular in relation to hormonally active chemicals [2] which are endocrine disruptors (EDs) and/or which can produce the low-dose effect. The above can take place during exposure to low concentrations of undesirable substances in feeds which can produce U-shaped or reverse U-shaped (hormesis) non-monotonic dose-response (NMDR) curves [30]. The NMDR hypothesis suggests that an ambiguous dose-response relationship does not support direct monotonic extrapolation or meta-analysis of the risk (clinical symptoms or laboratory results) associated with exposure to high and low doses of mycotoxins [11].

The concept of the lowest dose (unrelated to homeopathy), which produces an effect opposite to our expectations, is garnering interest in biomedical practice. For this reason, a thorough knowledge of the mechanisms responsible for the final outcome is required for effective decision making [33,34]. Endocrine disruptors have undermined traditional concepts in toxicology, in particular the “dose makes the poison” assumption because when administered in low doses, EDs induce changes that are not observed at higher doses [2]. Despite the above, numerous studies into natural hormones and EDs, including ZEN, demonstrated that low doses produce ambiguous responses [4,35].

The simplest method of determining the dose should involve the identification of the lowest concentration of food-borne mycotoxins that provoke changes in the animals’ intestines [36]. The provisional maximum tolerable daily intake (PMTDI) is still ambiguous for most mycotoxins. As a result, food and health protection agencies have to adjust PMTDI values to account for the rarely occurring toxic or lethal effects and, most importantly, the neutral effects of mycotoxin poisoning which are most common.

It should also be noted that many mycotoxins are characterized by specific levels of toxicity, which does not imply that every exposure to those substances has toxic consequences [37]. The risk posed by a specific dose is determined by the relationship between the ingested concentration of an undesirable substance and its toxic effect. This effect is manifested only when the ingested dose is sufficiently high. The dose or the amount of the ingested substance determines its biological effects [29,38,39,40,41]. Low mycotoxin doses (equal to or smaller than the highest NOAEL value) can promote development and improve physiological functions through their stimulating influence, which indicates that mycotoxins do not exert negative effects when administered in quantities smaller than the threshold dose. The correlation between the ingested dose or concentration of a substance and the observed biological effect is known as the dose-response relationship [40].

The NOAEL dose is used to describe the dose-response relationship when a statistically or biologically significant increase in the frequency or intensity of harmful effects is not observed relative to control. The NOAEL dose also denotes an experimentally derived value at which the frequency or severity of negative consequences does not increase in a population exposed to the substance in comparison with the control sample, or if such consequences are biologically or statistically non-significant. In toxicology, NOAEL represents a dose or a concentration of a substance (for example, a chemical substance) or a factor (such as radiation) which does not produce undesirable effects in the studied animals (absence of clinical symptoms of disease such as mycotoxicosis), whereas higher doses or concentrations deliver such effects [29]. As suggested by the cited authors, NOAEL values can be used to evaluate the risk associated with exposure to the evaluated mycotoxins.

For diagnostic purposes, further research is needed expand our knowledge of morphological and functional changes in the reproductive system of pre-pubertal bitches exposed to threshold (NOAEL) doses of ZEN over longer periods of time.

The aim of this review article is to present the effects of endogenous and/or exogenous steroid compounds on animals, documented by histological, immunohistochemical and endocrinological studies, to evaluate the influence of ZEN and its metabolites on ovarian and uterine function in pre-pubertal female dogs.

## 2. Exposure to Feed-Borne ZEN

### 2.1. Presence of ZEN in Feeds

Nutritionally balanced feeds for companion animals, including dogs, contain ingredients of plant origin. The results of our studies [26] and other authors’ findings [24] indicate that plant-based commercial feeds are often a vector for undesirable substances which may have harmful consequences for animals [7], including canids. ZEN is an exogenous factor, but not a xenobiotic. This mycoestrogen is particularly dangerous for monogastric mammals, where it is rapidly and easily absorbed after ingestion [7]. As an activating factor [42], ZEN contributes to the functional redundancy of estrogen receptors (ERs) with the relevant consequences.

### 2.2. Eryptosis—One of the First Consequences of Exposure to ZEN

To broaden our understanding of ZEN’s influence on pre-pubertal bitches, the relevant findings should be compared with the results reported by Jilani and Lang [43] in a study where mature human erythrocytes were exposed to the analyzed mycotoxin. In the cited study, eryptosis occurred even at low doses of ZEN. Eryptosis is suicidal death of non-nucleated erythrocytes due to oxidative stress [44], and it is equivalent to the apoptosis of nucleated cells, including granular cells [45]. In eryptosis and apoptosis, the stimulating factor is Ca^2+^, which has been confirmed by other authors, including Hénaff *et al.* [46].

Jilani and Lang [43] suggest that exposure to ZEN leads to the shrinkage of erythrocytes due to the stimulated flow of Ca^2+^ to cytosol. The above results in hyperpolarization of the cell membrane and the loss of Cl^−^ and KCl with osmotically obliged water. Circulating eryptotic erythrocytes are quickly removed from the bloodstream. This mechanism ensures that the percentage of eryptotic erythrocytes *in vivo* remains low even after stimulated eryptosis. Accelerated loss of eryptotic erythrocytes can be accompanied by anemia. Moderate stimulation of eryptosis can be fully compensated by simultaneous induction of erythrocytosis. The processes are accompanied by a decrease in mean corpuscular haemoglobin concentration (MCHC), mean corpuscular haemoglobin (MCH) and, in particular, mean corpuscular volume (MCV), which provides indirect evidence for eryptosis [47].

### 2.3. Disrupted Hormone Secretion during Exposure to ZEN

Zearalenone and its metabolites alpha-zearalenol (α-ZEL) and beta-zearalenol (β-ZEL) disrupt reproductive functions in animals [48,49] due to their similarity to E_2_. Alpha-zearalenol is the major ZEN metabolite in dogs [50] and pigs, whereas in other animal species (chickens, cattle and sheep), the predominant metabolite is β-ZEL with much lower levels of metabolic activity. ZEN activity is determined by metabolic processes in plants and animals and the immune status [3,18] of the reproductive system (resulting from changes in steroid hormone concentrations during maturation, the reproductive cycle or pregnancy) of exposed animals.

#### Involvement of Selected Steroid Hormones—Estradiol (E_2_) and Progesterone (P_4_)

The effects of low doses of ZEN have been studied in pre-pubertal bitches [50] and multiparous anestrous bitches [5]. Progesterone (P_4_) concentrations were low in both cases, which implies that physiological processes in the reproductive system of bitches are not disrupted when ZEN is ingested in amounts that do not exceed a stimulating dose or a tolerance dose (in line with the principles of hormesis—30). Progesterone levels increased in the first two weeks of exposure to ZEN, and much higher peripheral serum levels of P_4_ were observed in female dogs exposed to ZEN doses equivalent to 150% NOAEL, in particular after 3–4 weeks. The highest concentrations of α-ZEL and, subsequently, ZEN were also noted on the above dates. In pre-pubertal female dogs, P_4_ levels are generally very low, which does not support the biotransformation of ZEN to α-ZEL due to low dehydrogenase activity. Hyperestrogenizm in the ovaries provoked a 7-fold increase in the number of *m*RNA transcripts, but only for 3β-HSD. The above could increase the concentrations P_4_ [51], but without significant clinical implications. Zearalenone, α-ZEL and β-ZEL are substrates and endocrine disruptors which affect steroidogenesis in granular cells and increase P_4_ levels, which was also observed by other authors [3,4,5].

Estrogen (E_2_) plays a different role in the discussed processes because its concentrations in the peripheral blood vessels of female dogs increase only after 28 days of exposure [50]. Contrary to the hypothesis formulated by Van Cruchten [52], changes could be observed in the physiological sequence in which the concentrations of the analyzed hormones increase during puberty (before the first estrus), which could lead to false behavioural symptoms (false estrus, but only under exposure to doses above NOAEL), false maturation, *i.e.*, the initiation of estrus, and histological changes in oestrogen-dependent tissues in the ovaries [53] and the uterus [21,54].

Regardless of the degree of exposure, hyperestrogenizm in the uterus causes focal vascular congestion and extravasation in the lamina propria and the vascular layer of the uterine wall in the uterine horn proximal to the ovary. Such changes are observed during initial stages of exposure to ZEN, and they are maintained during monotonic exposure to NOAEL doses. Higher doses provoke significant vasodilation and vascular congestion, but only in the uterine horn proximal to the body of the uterus. The vascular layer in all segments of the uterus is wide, and its width increases proportionally to the ZEN dose. Those changes could obstruct diagnoses of uterine dysfunctions. The problem is exacerbated by the fact that clinicians are often not aware of the dangers associated with the presence of undesirable substances, including ZEN, in commercial feeds [24,26].

### 2.4. Involvement of Selected Hydroxysteroid Dehydrogenases (HSDs)

**Figure 1:**
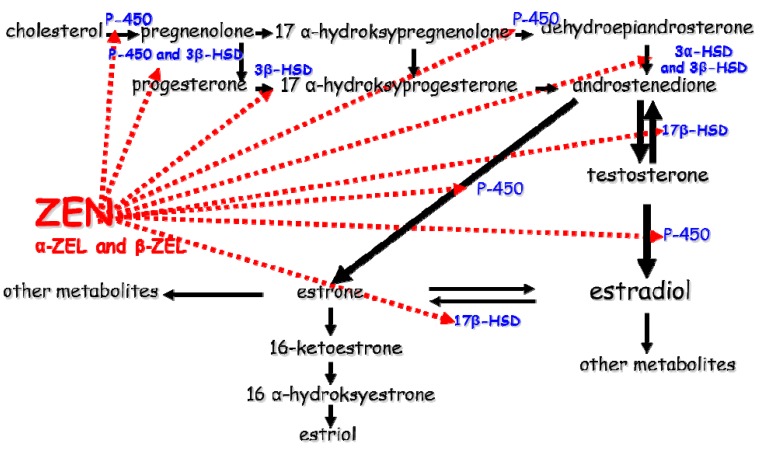
Sites where ZEN and its metabolites influence the activity of selected enzymes during steroidogenesis. ZEN, zearalenone; α-ZEL, α-zearalenol; β-ZEL, β-zearalenol; 3α-HSD, 3α-hydroxysteroid dehydrogenase; 3β-HSD, 3β-hydroxysteroid dehydrogenase; 17β-HSD, 17β-hydroxysteroid dehydrogenase; P-450, cytochrome.

Hydroxysteroid dehydrogenases play a major role in the metabolism, biosynthesis and deactivation of steroid hormones (Figure 1). Selected HSDs participate in metabolic processes of non-steroid compounds [55]. Hormones are synthesized and released by endocrine glands, they are transported by the circulatory system to target cells and/or tissues, and bind to specific receptor proteins (endocrinology). Labrie *et al.* [56] introduced the term “intracrinology” to describe “the cell-specific formation of estrogens and androgens with no significant release of active sex steroids in the circulation”. In target tissues, HSDs can modulate transactivation (transcription activation) of steroid hormone receptors or other extragenomic elements of a signal-transduction pathway. They act as molecular switches that modulate the activity of steroid hormone prereceptors [6].

The metabolism of natural environmental estrogens, including mycoestrogens, is often regarded as a detoxification process that lowers the concentrations of the parent substance (in this case, ZEN), but also produces new substances characterized by much greater toxicity (e.g., α-ZEL) than the parent compound [4]. The results of *in vitro* and *in vivo* studies indicate that ZEN reduces the activity of many steroidogenic enzymes of the cytochrome P-450 superfamily and the activity of HSDs, such as 3β-HSD and 17β-HSD and their isomers, which participate in the conversion of pregnenolone to P_4_ or estrone to E_2_ [51]. Those enzymes protect tissues and cells against harmful and undesirable substances, but they can also exacerbate the toxic effects of those substances, including ZEN and its metabolites, through the catalyzed changes [13].

In a study by Gajęcka *et al.* [51], biotransformation of ZEN in female dogs led to a 7-fold increase in the number of *m*RNA transcripts, but only for 3β-HSD. The above is accompanied by a 2-fold increase in *m*RNA levels for the CYPscc gene, which probably blocks final reactions in steroidogenesis. The exposure to ZEN at doses higher than NOAEL led to a significant increase in P_4_ levels.

Low enzyme activity in the first stage of detoxification resulted in energy deficits [18,49], which is also an important consideration for antiport activity in enterocytes [24,57]. The detoxification balance can be disturbed between the first and second stage of the process, subject to the degree of ZEN exposure. The above facilitates the release of metabolites, such as α-ZEL and β-ZEL, which significantly change the activity of steroidogenic enzymes (3β-HSD and 17β-HSD), depending on the substrate dose. In growing animals, in particular in livestock [3], α-ZEL destabilizes proliferative processes at tissue level [6].

The discussed processes are accompanied by abnormal values of the carry-over factor in peripheral blood in successive weeks of exposure. The above can be attributed to the fact that most phytoestrogens and mycoestrogens inhibit enzyme activity [55] by reducing P_4_ concentrations to the IC_50_ value. For this reason, mycoestrogens can affect not only the activity of steroid metabolizing enzymes, endogenous estrogens and androgens at the site of their synthesis, but also the activity of P_4_ in peripheral tissues [6,54,58]. The simultaneous decrease in α-ZEL levels results from: (i) an increase in the concentrations of the substrate (ZEN) available for 3β-HSD; or (ii) inhibited activity of 3β-HSD oxidase due to excessive amounts of the substrate [59].

**Figure 2:**
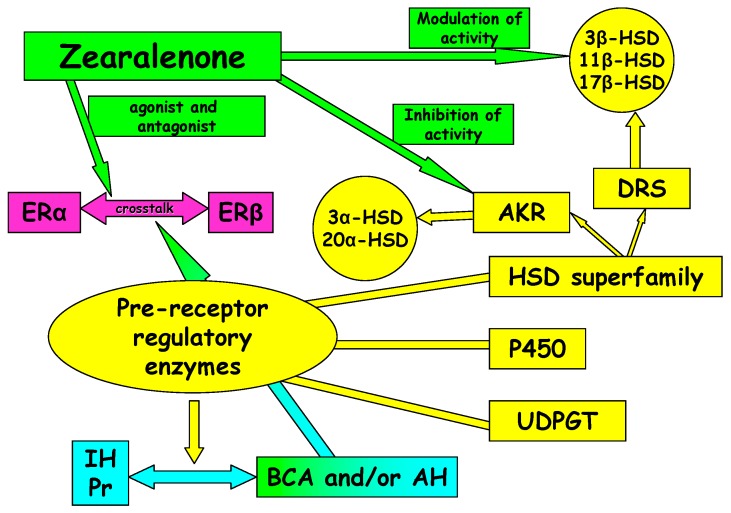
Estrogen receptors and the activation of pathways involved in rapid estrogen signaling. IH, inactive hormone; Pr, precursor; BCA, biologically active compound; AH, active hormone; HSDs, hydroxysteroid dehydrogenases; AKR, aldo-keto reductase superfamily; DRS, dehydrogenase/reductase superfamily; UDPGT, uridine diphosphate glucuronosyltransferase; 3α-HSD, 3α-hydroxysteroid dehydrogenase; 3β-HSD, 3β-hydroxysteroid dehydrogenase; 11β-HSD, 11β-hydroxysteroid dehydrogenase; 17β-HSD, 17β-hydroxysteroid dehydrogenase; 20α-HSD, 20α-hydroxysteroid dehydrogenase; P450, cytochrome P450.

17β-HSDs actively participate in final stages of steroidogenesis (Figure 1) [58]. Exogenous substrates act as non-steroid ligands or steroids, and as strong electrophiles, they can bind to the alkyl group and permanently inhibit enzyme activity. Estrogen activity is controlled by ER expression and modulation in the endoplasmic reticulum resulting from mutual conversion between hormones (e.g., E_2_) and their inactive counterparts (E_1_ and estrone sulphate). 17β-HSDs are of great significance in both pathways because they activate E_1_ to E_2_ and deactivate E_2_ to E_1_, and they are the major enzymes to be released during the development, growth and function of reproductive tissues [58]. The presence of 17β-HSDs is conditioned by the expression of tissue-specific compounds for ERs. Variations in the expression of these enzymatic prereceptor regulators (Figure 2) can increase the concentrations of E_2_. In the studies by Gajęcka [60] and Gajęcka *et al.* [50], an increase in E_2_ concentrations, an absence of ERα and a decrease in ERβ levels in the ovaries were inversely proportional to the ZEN dose. Their findings were consistent with the results of earlier studies.

## 3. Oestrogen Receptors

### 3.1. Ovaries

In the ovaries of sexually mature mammals, such as pigs, rats, hamsters and mice as well as humans, ERs are found mainly in granular, theca, stroma, luteal and paraluteal cells of the luteum and surface epithelial cells [42]. According to most researchers, ERβ is the major estrogen receptor in sexually mature females [16]. In a study by Couse *et al.* [61], the ratio of ERβ to ERα *m*RNA levels in the ovaries of pubertal rats was determined at 5:1, which confirms that ERβ is the predominant ER in sexually mature females of the investigated species. In female dogs, the presence of ERβ was demonstrated at each stage of ovarian follicular development, from the primordial follicle to the ovulatory follicle [60]. ERα were found only in large pre-ovulatory follicles and in early developmental stages of the corpus luteum in pigs [16] and in the uteri of female dogs [21]. Similar patterns of ERα *m*RNA expression in the luteal phase in the ovaries of bitches were reported by Hatoya *et al.* [27]. The above authors did not observe ERα *m*RNA expression in oocytes or cumulus oophorus cells during the development of ova in the oviducts, a process that would normally be accompanied by an increase in E_2_ concentrations.

By binding to ERs, ZEN initiates conformational changes that promote binding to estrogen response elements (EREs) in DNA, leading to the agonistic/antagonistic transcription of estrogen-sensitive genes. The resulting balance is determined mainly by the type of ERs in the analyzed tissue and the relative abundance of ligands or ERs. ERα and ERβ ligand binding domains have a very similar structure, and many ligands bind to both receptors with equal affinity. In phytoestrogens and mycoestrogens, ER transcription can be activated already at concentrations of 1–10 nM. Zearalenone’s affinity for ERs in target cells and tissues is estimated at 1%–10% in comparison with E_2_ [59]. The activity of ZEN and ZEL in target tissues is also determined by the type of ERs. In human cells, ZEN demonstrates greater affinity for ERα than ERβ, and it is a strong agonist of ERα [42]. In *in vitro* studies of human cells, ZEN’s affinity for ERβ varied subject to its concentration. When ZEN demonstrated greater affinity for ERβ than ERα, α-ZEL bonded more actively to ERα than ERβ, and *vice versa*. The degree (%) of bonding is always much lower than when E_2_ binds to ERs [60].

The above indicates that in canine ovaries, E_2_ concentrations decrease in the absence of ERα and the presence of only ERβ. Monomeric ERs influence constitutive gene expression without the involvement of estrogen. This applies particularly to ERβ which binds specifically to DNA at EREs [62], which is crucial for development. This implies that ovarian cells can develop despite very low concentrations of E_2_, but the presence of ZEN decreases ER levels [60].

ERs also influence epigenetic modification mechanisms [63]. In this process, gene activity in the cell nucleus is modified to “regress” the cell to an earlier developmental stage. da Silva Faria *et al.* [64] demonstrated that maternal protein-energy malnutrition leads to morphological changes in the ovaries, increases serum E_2_ concentrations and decreases the *m*RNA expression of ERα, ERβ1 and ERβ2 in the female offspring of rats.

ERα and ERβ ligand binding domains have a very similar structure, and they bind to both receptors with equal affinity. However, many exceptions exist, including genistein, a phytoestrogen with 5-fold higher affinity for ERβ than ERα. Genistein is an example of a selective ER modulator. ZEN also belongs to this group [57].

Changes in uterine tissue could be provoked by ZEN and α-ZEL which bind to ERα. ERα levels in uterine tissue decrease with an increase in the dose of ZEN and other exogenous estrogens [65]. The above suggests that the extragenomic effects of estrogens could be responsible for vascular congestion in the horns and the body of the uterus [66].

### 3.2. Uterus

The distribution of ERs in the uterus suggests that the observed changes could be provoked by ZEN and α-ZEL which bind to ERα. [63,65,67]. ERα levels in uterine tissue decrease with an increase in the concentrations of feed-borne ZEN and other exogenous estrogens. Our knowledge of the distribution and levels of ERα during exposure to ZEN indicates that vascular congestion in the horns and the body of the uterus could result from extragenomic effects of estrogens [66], similarly to the effects described in the *Ovaries* section. As an exogenous ligand, ZEN induces an increase in E_2_ concentrations [45], which leads to vasodilation [68]. Contrary conclusions were formulated by Barton [42] who argued that vasodilation has to be accompanied by coexpression and cross-talk between ERs. Pathological states in the uteri of female dogs exposed to ZEN can be thus provoked by intensified vascular congestion in the lamina propria and vascular layers in the uterine horns. The above results from angiogenesis, a specific type of neoangiogenesis, or vasodilation [68]. The associated consequences were discussed in the *Histopathology of selected reproductive structures–ovaries* section. The presence of ERα in the uterus supports the maintenance of normal E_2_ levels in female dogs not exposed to ZEN. Monomeric ERs influence constitutive gene expression without the involvement of estrogen [62], which implies that uterine cells can develop despite very low concentrations of E_2_ [55]. The presence of steroid-like compounds such as ZEN decreases ER levels due to functional redundancy caused by excessive concentrations of substances of various origin.

## 4. Metabolic Activity

### 4.1. Ovaries

The degree of ovarian cell apoptosis can be determined in the TUNEL assay [69]. This method supports the identification of cells with characteristic features of apoptosis, including cells in early stages of suicidal death without clear morphological changes [70]. In most studies, the presence of apoptotic cells is determined with the use of the apoptotic index (AI) which describes the percentage of apoptotic cells in 1000 analyzed cells [71].

In one of the reviewed articles, the AI median in the ovaries of 30 female dogs was determined at 13 [72]. The AI median increased to 13.45 under exposure to a lower ZEN dose and to 17.84 under exposure to a higher ZEN dose, compared with 8.59 in the control group. The reported results are difficult to compare with other authors’ findings, and it can only be assumed that the noted values are high and indicative of intensified apoptosis in the ovarian cells of pre-pubertal bitches exposed to ZEN. The reported AI values were higher than in the oocytes of pre-pubertal cattle where they were determined at 7. In sexually mature animals, median AI values can be as high as 23. The apoptotic index increases with age, therefore, physiological AI values in pre-pubertal bitches should be low.

Tatay *et al.* [73] and Wang *et al.* [74] conducted *in vitro* studies of human cells and ultrastructural studies of follicular cells in bitches exposed to ZEN [72]. The cited authors observed degeneration processes in oocytes, primordial ovarian follicles and developing cells. Complete degeneration of oocytes was reported in most cases. Follicles were filled with cellular debris composed of remnant mitochondria, endoplasmic reticulum, vacuolized nuclei and cytosol. Those observations indicate that ZEN and α-ZEL are characterized by significant cytotoxicity even at low concentrations.

A loss of cell junctions, vacuolization of intercellular spaces, loss of major cytoplasmic organelles and, most importantly, unclear contour and complete obliteration of the internal structure of mitochondria were observed in follicular cells. Mitochondria are responsible for maintaining the required energy (ATP) levels, and energy shortage leads to apoptosis [75]. All cells have processes on apical and lateral surfaces.

Oocytes and follicular cells are connected by gap junctions throughout the entire period of growth and development. In a study by Yang and Rajamahendran [76], apoptotic changes of unknown aetiology were much more frequently observed in bovine oocytes where gap junctions were loosened in successive stages of embryonic development after *in vitro* fertilization. Therefore, the questions arises whether ZEN and its metabolites could be responsible for the loosening of gap junction structure. It seems possible since the loss of gap junctions and enlarged intercellular spaces were observed in female dogs exposed to near NOAEL doses of ZEN [72]. In other studies, the morphology of follicular cells and follicular walls was not directly correlated with oocyte quality. Ovarian follicles generally contain a certain number of follicles with apoptotic changes, and the apoptotic signal is transmitted from follicular cells to oocytes only when critical activity levels have been exceeded, for example, when ATP concentrations decrease due to mitochondrial damage [75] or natural processes [10,72].

In evaluations of metabolic profiles of tissues, cell proliferation can be analyzed by the PCNA (proliferating cell nuclear antigen) test. Mitotic proliferation is one of the mechanisms that maintain homeostasis. The proliferation index (PI) of the PCNA is determined in the test [71]. Proliferative activity of ovarian follicles was less expressed in female dogs exposed to ZEN [72]. The PI median was determined at 25.49. In female dogs not exposed to ZEN, the PI median reached 35.49, which indicates that proliferation processes are significantly slowed down in the oocytes of primordial and growing follicles under exposure to ZEN. Significant differences in proliferative activity levels were not reported in atretic follicles. Proliferative activity decreased in response to long-term exposure, and the described changes are also symptomatic of hyperestrogenizm [73]. Proliferation was clearly intensified in female dogs not exposed to ZEN. Their ovarian structures were healthy, which points to normal proliferation and normal estrogen secretion (very low levels) by follicles [77].

In female dogs exposed to ZEN, apoptotic processes in ovarian cells were induced inside cells and began in the mitochondria [73]. The presence of ZEN and/or its metabolite α-ZEL provokes hyperestrogenizm, which increases mitochondrial concentrations of Ca^2+^ at cellular level. Endogenous and exogenous estrogens increase the levels of cellular Ca^2+^, thus activating mitochondrial protein phosphatase, which leads to the dephosphorylation of cytochrome c oxidase. Active protein contributes to an increase in mitochondrial membrane potential and concentrations of Ca^2+^, but the mechanism responsible for the above processes has not yet been fully elucidated [78]. An increase in the mitochondrial levels of Ca^2+^ promotes the production of free radicals which induce apoptosis [75]. In view of the characteristic properties of endogenous and exogenous estrogens, it can be assumed that exposure to above NOAEL doses of ZEN produces large calcium deposits in the mitochondria, cellular debris and oocyte remnants.

Ultra-histological studies [45] demonstrated that cytosolic Ca^2+^ levels increase when nucleated granular cells are stimulated, but the observed increase is not evenly distributed. It is particularly visible near calcium channels in the plasma membrane and in the endoplasmic reticulum which releases Ca^2+^ [78]. Mitochondrial Ca^2+^ buffering not only influences the calcium signal (amplitude, oscillation frequency), but it can exert a protective effect on cells. When those protective effects are weakened, organelles are damaged and apoptotic or even necrotic processes are initiated in cells or tissues. This is particularly important when cell stimulation leads to a high local increase in Ca^2+^ levels or Ca^2+^ blockage. In the cited experiments, a very high local increase in Ca^2+^ concentrations was required for secretion in sexually mature individuals.

The presence of Ca^2+^ in the mitochondrial matrix could be attributed to: (i) uniport concentration; and (ii) Ca^2+^ blockage observed in hyperestrogenizm. Physiological concentrations of Ca^2+^ in the mitochondrial matrix should change in response to fluctuations in cytosolic Ca^2+^ levels. The concentrations of Ca^2+^ have to be increased to regulate mitochondrial EDs which maintain hormonal homeostasis at the prereceptor level. Zearalenone is also a parent substance for HSDs [78], as discussed in the section titled *Involvement of selected hydroxysteroid dehydrogenases*.

### 4.2. Uterus

In a study by Stopa *et al.* [21], the highest AI values in the uterine structures of female dogs exposed to ZEN were reported in: (i) lamina propria; (ii) muscularis mucosa; and (iii) epithelium. The lowest AI values were reported in uterine glands. The above results indicate that exposure to ZEN led to the loss of glandular activity in the uterine horn proximal to the ovary, which coincided with the loss of ovarian activity [53]. There was a predominance of coil-shaped uterine glands, whereas spiral-shaped glands were observed only in the fundal layer of shallow uterine glands, which is characteristic of young animals. Extensive gland-free areas were also noted. No differences between ZEN-exposed groups were observed in the uterine horn proximal to the body of the uterus. In the body of the uterus, low levels of glandular cell activity were observed in all cases.

The described situation points to abnormal development of the uterine wall in newborn female dogs. Uterine adenogenesis begins at the end of the first week of life. From that moment onwards, epithelial and stromal cells proliferate less intensely. Adenogenesis is generally completed at the age of 6 to 8 weeks, which is when ERs are stimulated in the uterus [77]. The presented results [21] were observed in 16-week-old bitches that were exposed to ZEN in the preceding 6 weeks when adenogenesis should have been completed. Female beagles reach sexual maturity at around 6 months of age [22], therefore, the examined dogs would reach reproductive maturity in another 4–8 weeks. On the day of the analysis, secretory cells and all structures of the uterine mucosa should be inactive. The observed immunohistochemical and histopathological changes in female dogs exposed to ZEN are similar to the changes noted during the commencement of late anoestrus in multiparous bitches, but not in pre-pubertal female dogs. Regardless of the dose, exposure to ZEN induced apoptotic process in the uterus. The above could probably be attributed to the continued presence of ZEN, α-ZEL and β-ZEL in dogs throughout the experiment [50].

Exposure to low doses of ZEN increases the production of steroid hormones, in particular P_4_, in initial stages of the experiment [50]. The concentrations of E_2_ increase only towards the end of exposure despite the accompanying ovarian atresia [72]. Contrary to the hypothesis formulated by Van Cruchten [52], changes could be observed in the physiological sequence in which the concentrations of the analyzed (before the first estrus [79]). Those changes, commonly referred to as a hormones increase during puberty “hormonal imbalance” [2], could lead to false behavioral symptoms (false estrus), false sexual maturation (morphotic changes in the uterus) or histological changes in estrogen-dependent tissues (increased secretory activity of uterine glands) [21,63]. Those symptoms could prevent accurate diagnoses of the reproductive tract (uterus) before estrus.

Zearalenone and its metabolites disrupt the hormonal balance by inducing conformational changes, similarly to E_2_, and binding to ERs [16]. This leads to histological changes in the ovaries [53] and the uterus [21,63,80]. According to the literature, ZEN and its metabolites are agonists of ER-α (found mainly in the uterus, but not in the ovaries—27, 60) and partial antagonists of ER-β. Gajęcka *et al.* [50] demonstrated that ZEN and α-ZEL are endocrine disruptors (EDs) [2] which initiate the uncontrolled release of steroid hormones (P_4_ and E_2_) in various stages of prepubertal development [3,4].

The described functional imbalance leads to simple glandular hyperplasia [65] in the uterine horn proximal to the ovary [21]. This initial secretory stage is accompanied by the growth of glandular cells, stromal cells and coiling of uterine glands in female dogs exposed to ZEN. The above increases the number of glands in dogs exposed to a lower ZEN dose and decreases the number of glands in bitches exposed to a higher ZEN dose, relative to the control. In dogs exposed to ZEN, the secretory activity of glandular cells was weaker in comparison with the control group where an average number of glands and moderate levels of secretory activity were reported. In dogs exposed to ZEN, the PI values in the lamina propria increased in all analyzed regions of the uterus, whereas the PI values in uterine glands increased only in the uterine horn proximal to the body of the uterus. Similar results were reported by Gropetti *et al.* [79].

The results presented by various authors [60,63,65,67] suggest that exposure to ZEN also promotes extragenomic effects of estrogens [66]. As an exogenous ligand, ZEN induces an increase in E_2_ concentrations [45], which leads to vasodilation. Contrary conclusions were formulated by Barton [42] who argued that vasodilation should involve coexpression and cross-talk between ERs. The results of histopathological [21] and immunohistochemical analyses of uterine tissue indicate that the above could be attributed to: (i) intensive vascular congestion of the lamina propria and vascular layers of the horn of the uterus in female dogs exposed to ZEN; or (ii) angiogenesis, a specific type of neoangiogenesis, or vasodilation. Those mechanisms lower blood pressure because the total volume of the circulatory system increases when blood volume is stable. The flow of blood through uterine tissue is slowed down, which provides ZEN and α-ZEL with easier access to estrogen-dependent cells that contain ER proteins. Monomeric ERs influence constitutive gene expression without the involvement of estrogen [62]. Therefore, uterine cells can develop despite very low concentrations of E_2_. ZEN decreases ER levels due to functional redundancy caused by excessive concentrations of estrogen-like substances of various origin.

The activity induced in the lamina propria and uterine glands could also be attributed to hormesis, a biological phenomenon whereby toxin doses below NOAEL (threshold) values have a stimulatory/adaptive effect on the exposed organism, but produce an inhibitory effect when administered at doses above NOAEL [81]. Such conclusions can be formulated based on the results reported by Calabrese [30].

## 5. Histopathology of Selected Reproductive Structure

### 5.1. Ovaries

**Figure 3:**
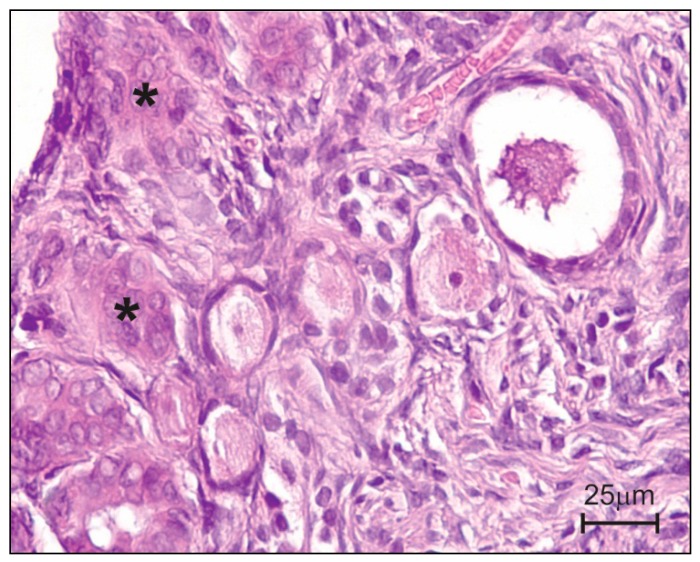
Histological structure after experimental administration of low doses (NOAEL) of ZEN to pre-pubertal bitches [53]. Invaginations of surface epithelium into connective tissue (asterisks).

In female dogs, ovaries are covered by “ovarian surface epithelium” (OSE—Figure 3) which plays various physiological roles: (i) it acts as a barrier for bioactive diffusion between the ovarian stroma and OSE; and (ii) it controls the periodic release of mature and developing ovarian follicles. In a study by Gajęcka *et al.* [50], OSE histology revealed the presence of epithelial cells in other ovarian structures. Small epithelial inclusion glands were observed under the tunica albuginea, whereas in other animals, the changes were more pronounced, and the noted invaginations reached the external cortex. The invaginations were probably produced in response to ZEN and α-ZEL, and their size and occurrence frequency were directly proportional to ZEN doses ingested with feed and to the period of exposure. The above was observed in sexually mature bitches (before ovulation), where it induced physiological apoptosis [82]. In pre-pubertal bitches, this is a pathological condition because primordial follicles are produced between the age of 17 and 54 days [83]. Primary follicles are observed in the course of 120 successive days, whereas the described changes took place at the age of 70 to 112 days.

Those changes can be provoked by intensified vascular congestion due to a higher number of capillaries and/or vasodilation. Both conditions lower blood pressure because the total volume of the circulatory system increases when blood volume is stable. The flow of blood through ovarian tissue is slowed down, which provides ZEN and α-ZEL with easier access to estrogen-dependent ovarian cells, as discussed in the *Metabolic activity–Ovaries* section.

In female dogs exposed to mycotoxins, the observed increase in the number of atretic follicles was directly proportional to the ZEN dose ingested with feed [20]. Most follicles were located in the medulla, therefore, it can be assumed that follicular atresia was not spontaneous (the evaluated bitches were very young), but that it was induced in larger follicles. This hypothesis is supported by the fact that female dogs were exposed to ZEN over a period of 42 days, and histological analyses was performed on samples collected on the last day of the experiment.

In larger follicles, atresia began in the follicular wall, *i.e.* from the exterior, as connective tissue penetrated the follicles. Under exposure to ZEN, theca cells began to resemble paralutein cells, becoming a structural element of the interstitial gland and an additional source of estrogens, which was confirmed by the hormonal imbalance observed in female dogs exposed to ZEN [50].

### 5.2. Uterus

Maturing ovarian follicles induce physiological morphometric changes, including the growth of uterine mucosa. Morphometric changes can also take place in response to exogenous estrogens, such as ZEN, which induce hyperestrogenizm. Regardless of the degree of exposure, an increase in: (i) weight of the horns of the uterus; (ii) length of the body of the uterus; (iii) diameter of the horn or the body of the uterus; and (iv) height and thickness of mucosal folds was observed in all regions of the uterus proportionally to the ZEN dose [21].

A functional imbalance contributes to simple glandular hyperplasia in the uterine horn proximal to the ovary. According to Schlafer and Gifford [65], this condition marks the beginning of the secretory phase which is accompanied by the growth of glandular cells, stromal cells and coiling of uterine glands in bitches exposed to ZEN. The number of glands changes proportionally under exposure to NOAEL doses, whereas the reverse is observed when ZEN doses are above NOAEL.

In animals exposed to ZEN, Schlafer and Gifford [65] and Stopa *et al.* [21] observed hyperplasia in the uterine horn proximal to the body of the uterus. The number of uterine glands varied subject to the ZEN dose. A predominance of spiral glands reaching the muscularis mucosa was reported under exposure to NOAEL doses of the mycotoxin. At higher doses of ZEN, uterine glands were less numerous (within the norm) with a predominance of coil-shaped glands, whereas spiral glands were observed only in the fundal layer. The glands were characterized by low levels of secretory activity. Extensive gland-free areas were also noted.

Fibroblasts and fibrocytes, the most common cells of connective tissue in animals, are also effective indicators of uterine homeostasis in female dogs. Those cells regulate tissue homeostasis under physiological conditions. Fibroblasts are activated and differentiated in response to tissue damage, which induces the production of the extracellular matrix in the affected tissue [84]. Under exposure to ZEN, the differentiation of fibroblasts and fibrocytes is observed in all uterine regions. With an increase in the ZEN dose, differentiation is gradually inhibited in the uterine horn proximal to the body of the uterus [21]. The above observations indicate that ZEN and its metabolites influence the structural framework of connective tissue in intercellular spaces by controlling fibroblast attachment. The attachment rate is inversely proportional to ZEN concentrations.

## 6. Summary

**Figure 4:**
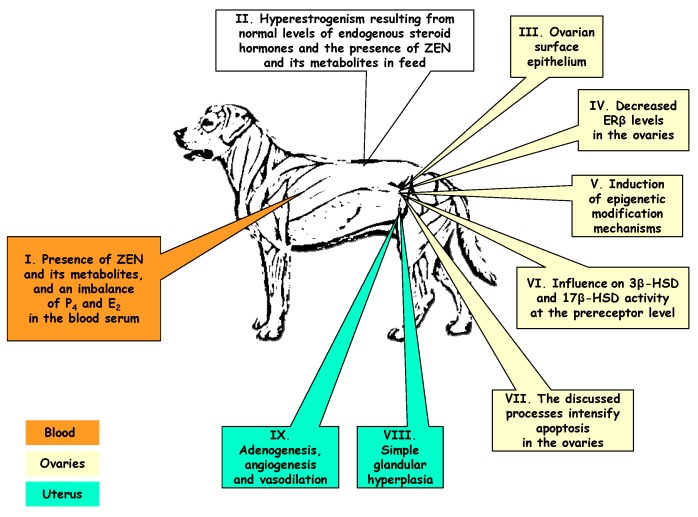
Preliminary determination of the diagnostic significance of selected analytical indicators of mycotoxicosis induced by zearalenone (at dose NOAEL) in bitches.

There is a general absence of studies that comprehensively analyze the influence of all diagnostic indicators, described in this review article, during long-term exposure of pre-pubertal bitches to NOAEL doses or higher doses of ZEN (Figure 4). The significance of individual indicators in various animal species has been discussed extensively, but separately, in the literature. This review paper analyses diagnostic indicators of the ovaries and uteri in pre-pubertal female dogs exposed to ZEN.

The observations made in this article support the conclusion that exposure to NOAEL doses or higher doses of ZEN induces hyperestrogenizm, while ZEN and α-ZEL concentrations in peripheral blood significantly influence the concentrations of P_4_ and E_2_ and modify the hormonal profile (Figure 4). Those processes are accompanied by atresia of ovarian follicles which are penetrated by connective tissue. This leads to the formation of indentations in ovarian surface epithelium which becomes an additional source of estrogens. Abnormal concentrations of P_4_ and E_2_ promote fibroblast differentiation in the uterus, which under long-term exposure to ZEN can lead to simple glandular hyperplasia accompanied by adenogenesis, angiogenesis and vasodilation with the relevant consequences, including morphometric changes. Those conditions clearly differ from the animals’ physiological state, and they are more visibly manifested in dogs that are exposed to lower ZEN doses. The observed changes may obstruct diagnoses of endometrial conditions, in particular in animals exposed to low mycotoxin doses. The presence of ZEN and its metabolites in peripheral blood and abnormal concentrations of steroid hormones decrease ERβ levels in the ovaries and induce epigenetic modification mechanisms which inhibit ovarian development in female dogs.

Zearalenone and its metabolites influence the activity of 3β-HSD and 17β-HSD at the preceptor level (Figure 2 and Figure 4). The dose of the substrate is inversely proportional to the noted effects. Zearalenone and its metabolites do not act selectively at the intracrine level because they inhibit 3β-HSD and 17β-HSD at doses above NOAEL. They can control HSDs and the metabolism of steroid hormones during exposure to mycotoxins.

In the ovaries, the discussed processes intensify apoptosis, slow down proliferation and cause adverse ultrastructural changes in primordial and growing follicles, in both oocytes and follicular cells. Apoptosis takes place in response to excessive Ca^2+^ concentrations in the mitochondria. Cell dysfunctions decrease or completely inhibit mitochondrial metabolic activity in oocytes, follicular cells and hilus cells.

Before the hormonal profile is modified, the presence of undesirable substances, such as mycotoxins, seems to be disregarded in initial stages of exposure due to food tolerance or compensatory response mechanisms. With time, the accumulation of mycotoxins in tissues can inhabit or accelerate cell proliferation in various bodily systems. Inhibited regeneration of the mucosa is an example of such a process.

## 7. Conclusions

A review of the available literature supports the formulation of the following conclusions regarding the exposure of pre-pubertal bitches to commercial feed contaminated with NOAEL or above NOAEL doses of ZEN:

- exposure to ZEN promotes changes in the metabolic profile of the reproductive system, which leads to adverse reproductive outcomes, such as follicular atresia,
- selected laboratory tests, such as the HSD activity assay, can be used to determine the presence of ZEN or predict pathological states in the affected tissues,
- modulation of HSD activity could be an effective measure to reduce the risk of ZEN intoxication,
- NOAEL doses of ZEN and its metabolites have a stimulatory effect on pre-pubertal bitches, whereas above NOAEL doses could inhibit vital life processes,
- the reviewed publications do not provide a clear answer as to whether the observed changes in the health status of animals are indicative of mycotoxicosis or result merely from exposure to ZEN.
